# *Burkholderia cepacia* complex and *Burkholderia gladioli* in large animals, dairy products, and livestock ticks: A systematic review of an emerging One Health concern

**DOI:** 10.1016/j.onehlt.2026.101647

**Published:** 2026-11-06

**Authors:** Zahra Baradaran-Seyed, Nader Mosavari

**Affiliations:** Razi Vaccine and Serum Research Institute, Agricultural Research, Education and Extension Organization (AREEO), Karaj, Iran

**Keywords:** *Burkholderia cepacia* complex, *B. gladioli*, Large animals, Host-associated patterns, Raw milk contamination, One Health

## Abstract

*Burkholderia mallei* is an established zoonotic pathogen that causes glanders, whereas *B. pseudomallei* is primarily an environmental pathogen that causes melioidosis in humans and animals. Most other *Burkholderia* species are environmental or plant-associated organisms; however, members of the *Burkholderia cepacia* complex (BCC) and *B. gladioli* are recognized opportunistic pathogens, particularly in susceptible humans. We conducted a PRISMA-guided systematic review of Web of Science, Scopus, and PubMed, supplemented by Google Scholar citation searching, through January 2025. We included eligible primary studies reporting naturally occurring infection, colonization, microbiome detection, or contamination involving large animals, dairy products, or livestock-associated ticks; we excluded animal-model and experimental-infection studies. Forty-eight unique studies met the eligibility criteria: 36 involving large animals, 10 involving dairy products, and two involving livestock-associated ticks. Reported organisms included *B. cepacia*, *B. cenocepacia*, *B. contaminans*, *B. vietnamiensis*, and *B. gladioli,* as well as *Burkholderia* identified only at the genus or complex level. Clinical disease was reported more often in horses and small ruminants, whereas findings in cattle and pigs were more often nonclinical or of uncertain clinical significance. BCC organisms and *B. gladioli* were also detected in raw milk and other dairy products, although detection frequencies varied substantially among studies and sample types. No study has demonstrated direct transmission from contaminated dairy products to human cases. The evidence was predominantly low-level and heterogeneous, indicating potential One Health relevance but precluding firm causal conclusions. Prospective surveillance, validated species-level identification, and genomic comparisons are needed to clarify pathogenicity, prevalence, and potential transmission pathways.

## Highlights

• Opportunistic *Burkholderia* were reported across large-animal host species.
• Clinical associations were more often reported in horses and small ruminants.
• Several clinical reports lacked documented host immunosuppression.
• BCC was found in dairy products; *B. gladioli* was reported in raw milk.
• Evidence was low-level; prospective surveillance and genomic studies are needed.

## 1 Introduction

The genus *Burkholderia* comprises diverse Gram-negative β-proteobacteria that inhabit a wide range of environmental niches, including soil, water, and plant-associated ecosystems (for current taxonomic information, see https://lpsn.dsmz.de/genus/burkholderia). Two species, *Burkholderia mallei* and *Burkholderia pseudomallei*, are major pathogens affecting humans and animals, causing glanders and melioidosis, respectively. *B. mallei* is an established zoonotic pathogen, whereas *B. pseudomallei* is primarily an environmental pathogen. Both species are listed as Tier 1 select agents because of their high pathogenicity and potential for deliberate misuse [1].

In contrast, many other *Burkholderia* species are primarily environmental or plant-associated organisms. However, several clinically relevant species, particularly members of the *Burkholderia cepacia* complex (BCC) and *B. gladioli*, are recognized as opportunistic pathogens. In human medicine, they are best known for causing severe, potentially life-threatening infections in susceptible individuals, especially patients with cystic fibrosis or chronic granulomatous disease [2]. Their intrinsic and acquired resistance to multiple classes of antimicrobials and disinfectants, combined with their ability to persist in pharmaceutical products and form biofilms, has led to numerous nosocomial outbreaks and product recalls [3], [4], [5], [6].

Sporadic reports over the past decades have described *BCC* and *B. gladioli* infections in livestock, including cases of mastitis, pneumonia, reproductive disorders, and septicemia in cattle, sheep, pigs, and horses [7], [8], [9], [10], [11], [12]. Nevertheless, these organisms are generally regarded as opportunists of limited veterinary importance and are often assumed to cause clinically significant disease primarily in animals with underlying conditions or impaired host defenses. BCC organisms have also been repeatedly detected in raw milk and unpasteurized dairy products in several countries, raising potential food safety concerns for susceptible consumers [8], [13], [14], [15].

To our knowledge, no systematic review has synthesized the available evidence on infection, colonization, microbiome detection, and contamination by non-*mallei*/non-*pseudomallei Burkholderia* species in large livestock species or comprehensively assessed their occurrence in dairy products and livestock-associated ticks. Consequently, it remains unclear whether risk assessments largely based on human clinical evidence adequately reflect their pathogenic potential and public health significance in a One Health context.

This PRISMA-guided systematic review was therefore undertaken to (i) document the reported occurrence and clinical manifestations of opportunistic *Burkholderia* spp. in large livestock species, including bovines, camelids, equines, swine, sheep, and goats; (ii) evaluate their detection in dairy products and livestock-associated ticks; and (iii) critically appraise the available evidence to inform future veterinary diagnostic, surveillance, and risk-assessment strategies.

## 2 Methods

### 2.1 Protocol and guidance

This systematic review was reported in accordance with the Preferred Reporting Items for Systematic Reviews and Meta-Analyses (PRISMA) 2020 statement [16]. The review protocol was not prospectively registered in a public database; however, we describe the methodology in full below to ensure transparency. We conducted the literature search in January 2025, focusing on opportunistic *Burkholderia* species in large-animal hosts, their associated vectors, and dairy products.

### 2.2 Search strategy

We conducted comprehensive database searches in Web of Science, Scopus, and PubMed. The search strategy combined two main concepts using the Boolean operator “AND.” The first concept targeted eligible *Burkholderia* members in the title, abstract, and keyword fields. The second concept included animal species names and standard terms for animal populations, which were searched in all available fields. We used Google Scholar and the reference lists of selected articles for supplementary citation searching. We provide the complete database search strategies and supplementary search approach in Supplementary Table S1.

To ensure comprehensive coverage of relevant taxa, we considered *Burkholderia* species names listed in the List of Prokaryotic names with Standing in Nomenclature (LPSN; https://lpsn.dsmz.de/genus/burkholderia). Additionally, the dataset of bacterial human pathogens compiled by Bartlett et al. (2022) was consulted to identify established and putative *Burkholderia* species (For more info: https://github.com/padpadpadpad/bartlett_et_al_2022_human_pathogens) [17].

We merged the two lists to create Supplementary Table S1, which presents the current and previous names of 23 putative and established *Burkholderia* species, excluding *B. pseudomallei* and *B. mallei*. An expanded list of animal populations was also used to allow comparison of the frequency of large-animal studies with that of other animal groups (see Supplementary Table S1).

We also used the Medical Subject Headings (MeSH) terms “*Burkholderia*” AND “Animals” in PubMed. We did not restrict the search by language or publication date. We imported retrieved records into EndNote and removed duplicates. We screened titles and abstracts against the eligibility criteria.

### 2.3 Eligibility criteria

Studies were eligible for inclusion if they reported identifying opportunistic *Burkholderia* spp. listed in Supplementary Table S1 in large animals (i.e., bovines, camelids, caprines, equines, ovines, or swine), their parasites or vectors, or dairy products. The review encompassed case reports, case series, comparative studies, outbreak investigations, and observational studies describing naturally occurring infection, colonization, microbiome detection, incidental isolation, or contamination.

We excluded studies involving human subjects, wildlife, aquatic animals, small mammals, rodents, birds, or non-livestock species. Further exclusion criteria comprised studies employing animal models or experimental infections; studies limited to laboratory strains without eligible animal or dairy-product data; investigations focused on *B. mallei* or *B. pseudomallei*; studies of biotechnology, industrial applications, or in silico analyses; environmental samples (e.g., manure, compost, or soil); detection on body surfaces in the absence of a defined clinical, colonization, microbiome, or surveillance context; and review articles. Conference abstracts or other reports available only as abstracts were included when they presented unique primary data that could be extracted.

### 2.4 Data extraction, evidence classification, and synthesis

Two reviewers independently screened titles and abstracts against the eligibility criteria. Disagreements were resolved by consensus. We retrieved full texts of potentially eligible reports and independently assessed them. We used the reference lists of selected articles and Google Scholar's “related articles” feature to supplement the search.

From each eligible study, we extracted a standardized dataset, including reference details; animal population characteristics (species, age, and sex); reported frequency of *Burkholderia* detection; diagnostic methods; clinical findings; antimicrobial susceptibility profiles; underlying conditions; and geographic location.

To improve transparency and avoid ambiguity in numerical reporting, we adopted specific terminology throughout the review. We defined “record” as a database or citation entry, “report” as an individual published document or conference abstract, “study” as a distinct underlying investigation, and “isolate” as an individual bacterial strain recovered from a sample. Multiple reports describing the same investigation or isolate were counted as one study in the analytical totals. When a single study encompassed multiple animal species, it was counted once in the total number of studies but separately for each relevant host-species category in the descriptive summaries. Consequently, the sum of the host-species categories could exceed the number of unique studies.

We assigned evidence levels to each study according to the evidence hierarchy presented by the Northern Territory Health Library (https://library.health.nt.gov.au/EBP/gradingevidence) and summarized them in Supplementary Table S2. Because study designs, populations, and outcome measures varied substantially, we adopted a narrative synthesis approach. We summarized the findings descriptively in tabular format. A concise summary table highlighting key patterns by host species is presented in the main text (Table 1), while the complete large-animal dataset is provided in Supplementary Table S3. Quantitative meta-analysis was not feasible and was not attempted.

**Table 1:** Summary of *findings on Burkholderia cepacia complex and Burkholderia gladioli* in large animals, stratified by clinical relevance and evidence level.

Host species	Clinical studies	Nonclinical studies a	Total studies	Predominant *Burkholderia* species^b^	Main clinical presentations (in clinical studies)	Geographic distribution (countries/regions)	Evidence level (range)^c^	Summary interpretation
Cattle	[8], [15], [28]	[19], [20], [22], [25], [27], [34], [35]	10	*B. contaminans*, BCC	Clinical and subclinical mastitis	Europe (Italy, UK), Africa (Sudan), Asia (China), Oceania (Australia, New Zealand), North America (USA)	2a–5	Mostly commensal or opportunistic; primary disease uncommon but documented (a clinical mastitis outbreak).
Horses	[9], [10], [11], [28], [29], [33], [36], [39]	[35], [37], [38]	11	*B. cepacia*, *B. gladioli*	Respiratory disease, abortion/endometritis, penile prolapse, keratitis, endocarditis, glanders-like lesions	Europe (France, Italy, Poland, Portugal), Africa (Egypt, South Africa), the Middle East (Iran), North America (USA), Oceania (Australia)	4–5	Several predominantly case-based studies reported clinical disease; nonclinical fecal, oral, and conjunctival findings were also reported.
Pigs	[18, 30, 43, 44]^d^	[21], [23], [24], [26], [40], [41], [42], [45], [46]	12^d^	BCC	Respiratory isolate, endometritis-associated microbiota, and findings in colostrum-deprived SPF piglets	North America (USA), Europe (Germany, Romania, Bulgaria), Asia (China, South Korea), South America (Brazil)	2a–5	Most findings involved semen contamination or microbiome detection; the causal relevance of endometritis-associated findings and other clinical-context detections remains uncertain.
Sheep	[7], [12], [47]	[42]	4	*B. cepacia*, *B. vietnamiensis*	Mastitis (subclinical), visceral/cutaneous abscesses, melioidosis-like abscesses	Europe (Spain, Bulgaria), and the Middle East (Saudi Arabia, Iran)	4–5	Clinical disease or associated lesions were reported in three studies; one study reported incidental isolation in clinically healthy lambs with parasitic infection.
Goats	[48]	[49]	2	*B. cepacia*, *B. vietnamiensis*	Septicemia; visceral abscesses in a coinfected goat	Southeast Asia (Malaysia, Philippines)	4–5	Both clinical disease and asymptomatic nasal carriage have been reported; evidence is limited to a few cases.

a Non-clinical studies encompass colonization, microbiome detection, semen contamination, or incidental isolation from healthy animals.

b BCC, *Burkholderia cepacia* complex. Most species-level identifications belonged to BCC or *B. gladioli*; several studies reported only genus-, complex-, or clade-level assignments.

c Based on the evidence hierarchy presented by the Northern Territory Health Library, the range indicates the lowest and highest evidence levels among studies for each host species (see Supplementary Table S2).

d Two references [43], [44] focused on the same pig isolate and are therefore counted as a single clinical study.

## 3 Results

The broader animal-focused screening identified 168 reports relevant to animal populations (Fig. 1; Supplementary Tables S2–S4). Of these, 48 unique studies met the eligibility criteria for the systematic review: 36 involving large animals, 10 involving dairy products, and two involving livestock-associated ticks. The remaining 120 reports concerned non-large-animal populations and were catalogued separately in Supplementary Table S4. Because several large-animal studies included more than one host species, the sum across host-specific categories exceeded the number of unique large-animal studies, yielding 39 host-specific entries from 36 studies.

**Fig. 1:**
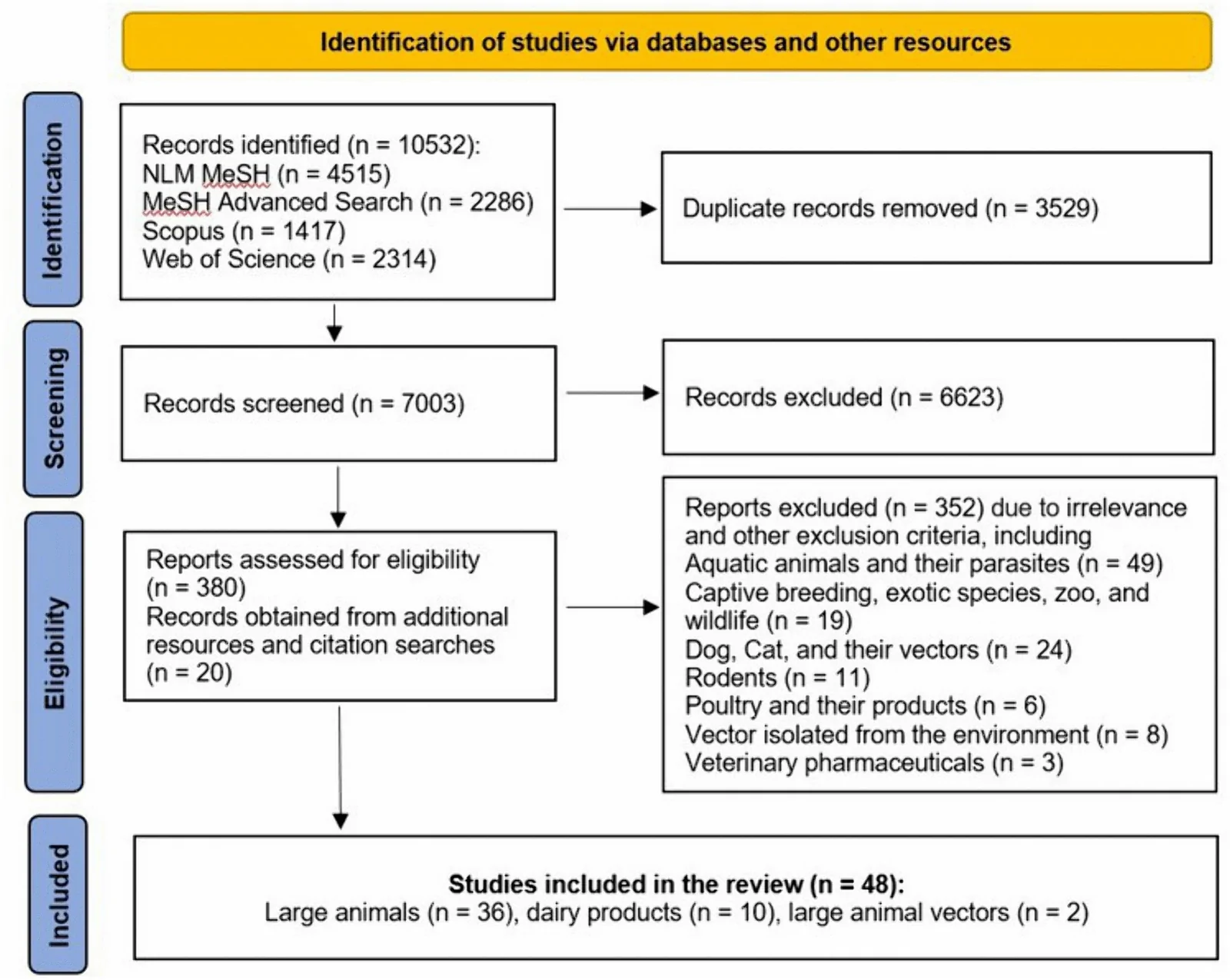
PRISMA 2020 flow diagram illustrating the systematic review process for opportunistic *Burkholderia* occurrence in large animals, livestock-associated ticks, and dairy products (PRISMA 2020 statement [16]). Google Scholar was used for supplementary citation searching; records retrieved through citation searching are included under additional resources.

### 3.1 Large animal studies

Table 1 concisely summarizes findings by host species, stratified into studies reporting clinical disease and those reporting nonclinical findings. Full bibliographic details for all cited references are provided in the reference list at the end of the article. Supplementary Table S3 presents the complete large-animal dataset, including diagnostic methods, clinical signs, antibiotic susceptibility profiles, and underlying conditions for each study.

Many large-animal records were individual case reports and case series. We identified only two clearly documented outbreak reports involving cattle mastitis [8] and sheep mastitis [7]. A comparative microbiome study also examined sows with endometritis [18]. Aside from a small number of controlled observational and trial studies graded as 2a and 3a–b [8], [18], [19], [20], [21], [22], [23], [24], [25], [26] and one 3c study [27], the remaining evidence fell within grades 4 and 5 (as detailed in Supplementary Table S2). Except for one conference abstract [28], all included records were peer-reviewed documents. The full texts of four studies could not be accessed despite extensive efforts [14], [29], [30], [31]. Most texts were published in English, except for two in Chinese [23], [30], one in Turkish [31], and one in French [29]; all had English abstracts. The earliest documentation of dairy products dates to the 1980s [14], [32], while the first records of large-animal cases date to the 1990s, particularly involving equine subjects with respiratory and cardiac insufficiencies [29], [33] and SPF piglets [30].

#### 3.1.1 Cattle

Ten studies documented *Burkholderia* in cattle [8], [15], [19], [20], [22], [25], [27], [28], [34], [35].

In one study of clinical mastitis, *B. contaminans* was the only pathogenic bacterium identified in the milk of 18 cows in a clinical mastitis outbreak in dairy cattle in China [8]. A retrospective analysis that included cattle reported BCC in samples from clinically infected animals [28]. In a trial involving cows with subclinical mastitis, inulin supplementation reduced the relative abundance of *Burkholderia–Caballeronia–Paraburkholderia* [15]. In the remaining seven studies, researchers detected *Burkholderia* in oral, ruminal, jejunal, fecal, or uterine samples, or isolated it from bile collected from clinically healthy slaughtered cattle.

#### 3.1.2 Horses

Eleven studies reported findings in horses [9], [10], [11], [28], [29], [33], [35], [36], [37], [38], [39]. All but three of these [35], [37], [38] identified *Burkholderia* from clinically ill animals or associated lesions. Clinical presentations included respiratory disease (tracheal aspirates) [10], vegetative endocarditis [33], abortion, and endometritis [9], penile prolapse with extensive infection [11], ulcerative keratitis [36], and a horse with glanders-like lesions that cross-reacted in serological tests for *B. mallei* [39]. A *B. gladioli* strain isolated from the gingival margin of a healthy horse displayed a broad resistance profile to multiple antimicrobial classes [38].

#### 3.1.3 Pigs

Twelve studies, published across 13 records, focused on pigs [18], [21], [23], [24], [26], [30], [40], [41], [42], [43], [44], [45], [46]. Four studies investigated *B. cepacia* contamination of extended semen; some reported reduced sperm motility, agglutination, or fertility-related effects [40], [41], [45], [46]. One study specifically investigated colostrum-deprived SPF piglets, in which *Pseudomonas cepacia* was isolated from normal and diarrheic feces and from dead piglets [30]. A comparative microbiome study found that *Burkholderia* was the dominant genus in vaginal samples from sows with endometritis at one of the two farms examined [18]. Two publications described the genomic characterization of a *B. contaminans* isolate obtained from the respiratory tract of a pig with respiratory disease [43], [44]. In the remaining studies, *Burkholderia* was detected in gastrointestinal, rectal, or vaginal microbiota of apparently healthy pigs, or was isolated incidentally from liver tissue of pigs with *Cysticercus tenuicollis* infection [21], [23], [24], [26], [42].

#### 3.1.4 Sheep

Four studies reported *Burkholderia* in sheep. Three studies documented clinical disease or associated lesions: a subclinical mastitis outbreak caused by *B. cepacia* genomovar III and *B. vietnamiensis* [7], cutaneous and visceral abscesses in an emaciated ewe [12], and melioidosis-like lung and liver abscesses detected during abattoir surveillance [47]. The remaining study isolated *Pseudomonas cepacia* from the liver tissue of clinically healthy lambs that were naturally co-infected with Cysticercus tenuicollis [42]. Although the parasitic cysts may have provided a niche for secondary bacterial colonization, the lambs showed no clinical signs of *Burkholderia* infection, and the finding was considered incidental.

#### 3.1.5 Goats

Two studies addressed cases in goats. One report described two dairy goats: *B. cepacia* septicemia was identified in one goat, while the other had concurrent *B. pseudomallei* and *B. cepacia* septicemia; the coinfected goat developed multiple visceral abscesses [48]. This review considered only findings related to *B. cepacia*. The other study identified *B. vietnamiensis* from a nasal swab of an apparently healthy goat in the Philippines [49].

### 3.2 Livestock-associated ticks

Two studies reported opportunistic *Burkholderia* spp. in hard ticks collected from large animals. In Mexico, researchers obtained 293 bacterial isolates from 100 individually processed ticks collected from cattle in Nuevo León and Zacatecas. *B. cepacia* was the second most frequent species (49/293, 16.7%) and the only *betaproteobacterium* identified from *Rhipicephalus microplus* (23/160 ticks, 14.4%) and *Otobius megnini* (26/117 ticks, 22.2%), but not from *Amblyomma cajennense* [50]. In Iran, analysis of the V6 region of the 16S rRNA gene from 39 adult *Ixodes ricinus* ticks collected from cattle and sheep in Mazandaran, Gilan, and Golestan provinces detected members of the *Burkholderia*–*Paraburkholderia* clade and *Burkholderia* sp. A3 (2016) alongside established tick-borne pathogens such as *Coxiella* and *Rickettsia* [51].

### 3.3 Dairy products

Ten studies published between 1986 and 2021 from seven countries evaluated the occurrence or survival of opportunistic *Burkholderia* spp. in raw milk, Swiss cheese, or homemade yogurt (see Supplementary Table S2). In South Africa, researchers identified 13.3% (6/45) of *Pseudomonas*-like isolates from raw milk as *B. cepacia* [32]. In the United States, *B. cepacia* was implicated as a major cause of rind rot in Swiss cheese because it produces excessive dimethyl disulfide [14]. In Türkiye, researchers recovered one *B. cepacia* isolate from 200 raw milk samples [31]; later, 17.9% (5/28) of homemade yogurt samples from Ağrı Province yielded *Burkholderia* spp., marking the first documented occurrence in yogurt [52]. In Northern Ireland, the highest reported prevalence was 53.8% (14/26) in raw bovine milk samples; all other foods and water tested were negative [53]. In a subsequent study, Moore et al. (2002) failed to detect *Burkholderia cepacia* complex in 21 bovine mastitis milk samples using a selective enrichment technique. HTST pasteurization completely inactivated BCC reference strains in milk experimentally contaminated with them [54]. In Finland, researchers identified six of 60 psychrotrophic isolates from refrigerated raw milk as *B. cepacia* and one as *B. vietnamiensis* [55]. In Egypt, 25.8% (31/120) of individual raw milk samples were positive for BCC (23.3% cows, 16.7% buffaloes, 33.3% sheep, 30.0% goats); 10 isolates were uniformly resistant to ampicillin and amoxicillin [13]. In Brazil, researchers recovered four *B. cepacia* and one *B. gladioli* among 128 Gram-negative psychrotrophic isolates from refrigerated raw milk and transport tanks [56]. In England and Wales (UK), 16S rDNA sequencing identified four *Burkholderia* spp. among bulk-tank milk isolates from 363 farms between 2015 and 2016 [57].

## 4 Discussion

### 4.1 Summary of main findings

This systematic review identified 48 unique studies of *non-mallei*/non-*pseudomallei Burkholderia* in large animals (*n* = 36; summarized in Table 1 and detailed in Supplementary Table S3), dairy products (*n* = 10; Supplementary Table S2), and livestock-associated ticks (*n* = 2; Supplementary Table S2). Most animal-associated records were case reports and laboratory investigations based on convenience sampling (evidence level 4–5), with few controlled observational studies, which are inherently prone to publication and diagnostic bias. Disease-associated findings included mastitis, endometritis-associated microbiome changes, respiratory disease, septicemia, abscesses, and abortion; however, causation remained presumptive in many reports. True incidence and prevalence remain unknown because population-based studies have not been conducted in large-animal species. Fig. 2 presents an integrated evidence map of host-specific clinical and nonclinical findings, detection in dairy products and livestock-associated ticks, and cross-cutting diagnostic and antimicrobial-resistance challenges.

**Fig. 2:**
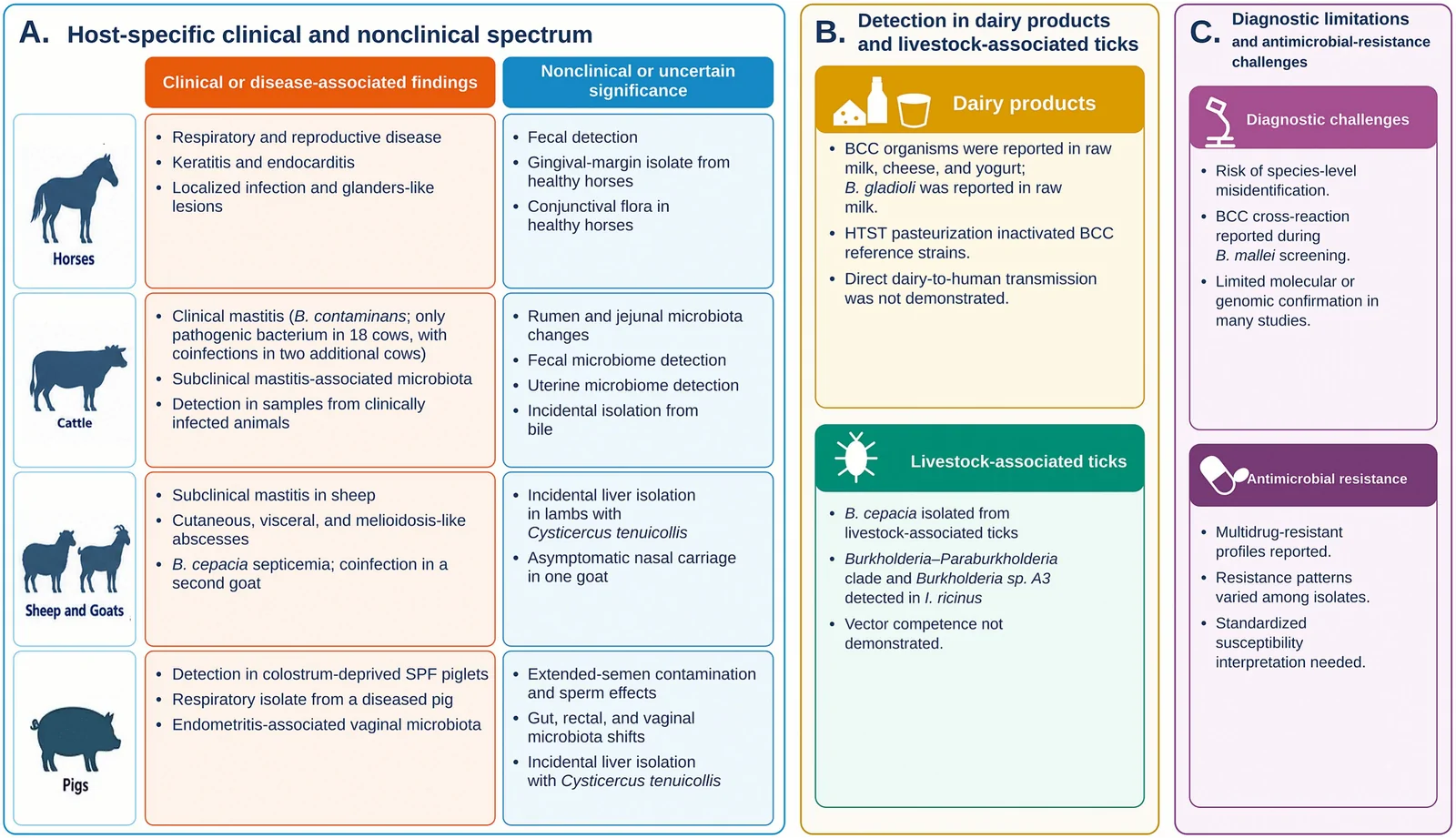
One Health evidence landscape of the *Burkholderia cepacia* complex and *B. gladioli* in livestock systems. (A) Host-specific clinical or disease-associated findings and nonclinical findings of uncertain significance in horses, cattle, sheep and goats, and pigs. Orange boxes indicate clinical or disease-associated findings, whereas blue boxes indicate colonization, microbiome detection, contamination, incidental isolation, or findings of uncertain clinical significance. (B) Detection in dairy products and livestock-associated ticks. Direct dairy-to-human transmission and tick vector competence were not demonstrated. (C) Diagnostic limitations and antimicrobial-resistance challenges identified across the included studies. The evidence was heterogeneous and largely low-level; therefore, the illustrated associations do not confirm causation or transmission. (For interpretation of the references to colour in this figure legend, the reader is referred to the web version of this article.)

### 4.2 Host-species-dependent patterns of association

Current knowledge of opportunistic *Burkholderia* spp. comes primarily from human medicine, where BCC and *B. gladioli* are recognized as serious threats, particularly in susceptible hosts, including patients with cystic fibrosis [2], [58]. As summarized in Supplementary Table S4, some reported infections in small animals and laboratory rodents occurred in animals with immunosuppression or underlying disease. Reports involving aquatic species, poultry, captive animals, and wildlife more often described microbiome detection, colonization, or environmental occurrence than confirmed primary disease.

This human- and small-animal-centric paradigm does not fully explain the patterns observed in large livestock species. This group of animals showed a marked host-species-dependent association pattern. Clinical disease was reported more often in horse and small-ruminant studies, whereas cattle and pig studies more often involved microbiome detection, contamination, or incidental isolation (Table 1; Supplementary Table S3). While this observation supports the hypothesis of a primary pathogenic role in these host species, it must be interpreted cautiously, as the underlying data are predominantly low-level. Comparative and controlled studies also detected opportunistic *Burkholderia* as components of gastrointestinal, reproductive, or milk microbiota without establishing a causal role [8], [19], [20], [21], [22], [23], [24], [25], [26].

Two well-documented mastitis outbreaks in sheep and cattle indicate that these organisms can act as primary pathogens under specific conditions [7], [8]. In both outbreaks, sampling of potential environmental or treatment-associated sources did not identify the outbreak organism, leaving the sources and transmission pathways unresolved [7], [8].

Detecting *Burkholderia* in parasitic or melioidosis-like lesions warrants further investigation [42], [47]. Abattoir surveillance detected *Burkholderia* in 14 of 90 sheep examined, highlighting the need for further research to assess the prevalence and clinical significance of this finding [47]. After the January 2025 search cutoff, Al Mamun (2026) reported bacterial growth in 8 of 30 samples from symptomatic goats in Bangladesh [59]. Of these eight culture-positive samples, five showed bacterial 16S rRNA amplification and two were positive by *groEL*- and *zmpA*-targeted PCR; phylogenetic analysis supported BCC identification. No bacterial growth was observed in 10 healthy controls. This post-cutoff study provides subsequent evidence of BCC occurrence in goats but does not establish causation or population prevalence. It was discussed only as contextual evidence and was not incorporated into the 48-study systematic-review dataset, Table 1, or the supplementary study counts.

The detection of non-*pseudomallei Burkholderia* in small ruminants with melioidosis-like lesions and the cross-reaction of BCC in a horse with glanders-like clinical signs highlight potential diagnostic challenges [39], [47]. In particular, the reported cross-reaction in glanders serologic testing suggests that opportunistic BCC organisms may complicate surveillance for *B. mallei* [39].

The substantial contribution of large-animal reports to the overall animal-related literature indicates that opportunistic *Burkholderia* spp. cannot be regarded solely as human- or laboratory-animal-associated opportunists and reinforces the need for dedicated veterinary surveillance and pathogenesis studies in livestock species.

### 4.3 Diagnostic challenges and species-level identification

The current study compiled a list of 23 established and putative species considered in the search strategy (Supplementary Table S1). However, many included studies did not identify *Burkholderia* at the species level or relied on conventional phenotypic methods with limited discriminatory resolution. Advanced molecular methods can improve the identification of BCC and *B. gladioli*, whereas conventional phenotypic systems may misidentify closely related organisms [6]. Jin et al. (2020) reported that commonly used approaches, including 16S rRNA, recA, hisA, and multilocus sequence analysis, may provide insufficient resolution for distinguishing some closely related BCC species [60]. They proposed that core phylogeny based on single-copy orthologous genes, combined with pangenome-based dDDH and ANI clustering, provides a more robust framework for species differentiation. MALDI-TOF MS may also support *Burkholderia* identification, provided that the spectral library contains appropriate reference spectra [60], [61], [62].

### 4.4 Dairy product contamination: Sources and public health implications

The dairy-product findings should be considered alongside reports of clinical and subclinical mastitis in livestock [7], [8]. Studies reported BCC organisms in raw milk, Swiss cheese, and homemade yogurt, whereas *B. gladioli* was reported in raw milk; detection frequencies varied substantially across studies and sample types [13], [52], [53], [56]. The reports did not identify whether animal infections or environmental factors during milking, storage, transportation, or processing into other products caused dairy contamination [52], [53], [54]. An experimental study found that HTST pasteurization inactivated BCC reference strains in milk contaminated under experimental conditions [54]. Given BCC's intrinsic multidrug resistance and its established role as a potentially life-threatening pathogen in susceptible individuals, consuming raw-milk products may pose a One Health food-safety concern. However, the available evidence does not establish a direct transmission chain from contaminated dairy products to human clinical cases or confirm homology between animal and human isolates. The findings presented here should therefore be interpreted as hypothesis-generating, highlighting the need for integrated molecular surveillance linking livestock, dairy products, and human populations.

### 4.5 Livestock-associated ticks: A potential vector role?

Ubiquitous saprophytic microorganisms commonly found in the environment may reside on animal surfaces. Two studies detected opportunistic *Burkholderia* in livestock-associated ticks from geographically distinct settings [50], [51]. These findings document bacterial presence in tick-associated microbiota but do not demonstrate mechanical or biological vector competence. Further studies using paired livestock, tick, and environmental samples, along with strain-level comparisons, are needed before inferring any transmission role.

### 4.6 Antimicrobial resistance and therapeutic challenges

The intrinsic and acquired resistance of BCC and *B. gladioli* to multiple classes of antimicrobials and disinfectants, coupled with their well-documented biofilm-forming capacity, poses significant challenges for both veterinary and human health [5], [6]. Among the reviewed studies, antimicrobial susceptibility testing, where performed (Supplementary Table S3), reported multidrug-resistant profiles in several tested isolates. For instance, a BCC isolate from a stallion with penile prolapse was resistant to ceftazidime, meropenem, tetracyclines, and trimethoprim-sulfamethoxazole, remaining susceptible only to ciprofloxacin, enrofloxacin, levofloxacin, and streptomycin [11]. Similarly, *B. cepacia* contaminants in boar semen showed broad resistance to β-lactams, aminoglycosides, macrolides, and tetracyclines [40], [46]. A *B. gladioli* strain from a healthy horse displayed 100% resistance to aminoglycosides, quinolones, macrolides, and sulfonamides [38]. These observations underscore the therapeutic challenges opportunistic *Burkholderia* pose in veterinary practice and the urgent need for species-specific interpretive criteria. Susceptibility methods, antimicrobial panels, and interpretive approaches varied among studies, limiting direct comparison of the reported results. Standardized testing, species-level confirmation, and transparent reporting of interpretive criteria are therefore needed to improve comparability and clinical relevance.

### 4.7 Limitations

Several limitations must be acknowledged. First, the predominance of low-level evidence (grades 4–5), mainly case reports and case series, limits causal inference, and population-based incidence and prevalence data were unavailable. Second, the review synthesized biologically distinct findings, including clinical infection, colonization, microbiome detection, incidental isolation, and contamination, creating a risk of overinterpreting detection as evidence of disease relevance. Third, species-level identification was frequently lacking or based on phenotypic methods with limited discriminatory accuracy. Fourth, we could not access the full texts of four included reports, potentially limiting data extraction. Finally, the protocol was not prospectively registered, and study heterogeneity precluded application of a single conventional risk-of-bias tool. Therefore, the evidence-level classification used to describe study-design strength should not be interpreted as a full, study-specific risk-of-bias assessment.

### 4.8 Knowledge gaps and future research directions

Prospective surveillance and comparative studies with appropriate control groups are needed to determine the frequency and clinical relevance of opportunistic *Burkholderia* detection and to distinguish infection from colonization, microbiome detection, incidental isolation, and contamination. Whole-genome sequencing and comparative genomics of livestock, dairy-product, environmental, tick, and human isolates could clarify whether related findings represent transmission, common environmental exposure, or unrelated strain populations. The potential mechanical or biological role of ticks should be investigated using paired livestock, tick, and environmental samples. Validated molecular assays, comprehensive MALDI-TOF MS reference libraries, and standardized antimicrobial susceptibility methods are also needed to improve species-level identification and interpretation of clinically significant isolates.

## 5 Concluding remarks and implications for practice

Non-*mallei*/non-*pseudomallei Burkholderia* have been reported in multiple large-animal species, dairy products, and livestock-associated ticks, but their clinical relevance varies substantially. Clinical disease was reported more often in horses and small ruminants, whereas findings in cattle and pigs were more commonly associated with microbiome detection, incidental isolation, or contamination. However, the heterogeneous, predominantly low-level evidence precludes firm conclusions about host-specific pathogenicity, incidence, or virulence. Furthermore, neither direct dairy-to-human transmission nor tick vector competence has been demonstrated.

Clinically significant isolates should undergo species-level identification using validated molecular or genomic methods, supported where appropriate by comprehensive MALDI-TOF MS reference libraries. Antimicrobial susceptibility testing should be considered while recognizing current methodological and interpretive limitations. Targeted, risk-based monitoring of raw and unpasteurized dairy products may also be appropriate where susceptible consumers could be exposed. Until stronger evidence becomes available, a cautious, species-aware approach to veterinary diagnosis, surveillance, and raw-milk safety remains warranted.

## Declaration of generative AI and AI-assisted technologies in the manuscript preparation process

During the preparation of this work, the authors used AI-assisted Grammarly for language polishing and grammar checking. After using these tools, the authors reviewed and edited the content as needed and took full responsibility for the published article.

## CRediT authorship contribution statement

**Zahra Baradaran-Seyed:** Supervision, Resources, Methodology, Data curation, Conceptualization, Writing – review & editing, Writing – original draft. **Nader Mosavari:** Validation, Resources, Data curation, Writing – review & editing.

## Funding

This research received no specific grant from funding agencies in the public, commercial, or not-for-profit sectors.

## Declaration of competing interest

The authors declare that they have no known competing financial interests or personal relationships that could have appeared to influence the work reported in this paper.

## Data Availability

This systematic review synthesizes data from previously published studies. This study generated and analyzed no new primary data. All data relevant to the review are included in the manuscript and its supplementary materials, and the sources are cited and referenced accordingly.

## Appendix A Supplementary data

Supplementary material 1 All supplementary tables are provided in a single navigable Excel workbook entitled “Supplementary Data File.” The workbook contains an introductory Navigator worksheet with internal links to the following tables: Supplementary Table S1. Current and previous names of putative and established opportunistic *Burkholderia* species and the complete literature search strategy. Supplementary Table S2. Overview of the 48 included studies: 36 unique large-animal studies contributing 39 host-specific counts, 10 dairy-product studies, and two livestock-associated tick studies. Supplementary Table S3. Detailed characteristics of the 36 included large-animal studies, including study populations, bacterial identification, diagnostic methods, clinical findings, antimicrobial susceptibility results, and geographic locations. Supplementary Table S4. Categorized index and complete bibliographic list of 120 non-large-animal reports identified in the broader animal-focused search.
